# Supplementary material for: SinglePointRNA, an user-friendly application implementing single cell RNA-seq analysis software
Source: PLoS One. 2024 Jun 18;19(6):e0300567. doi: 10.1371/journal.pone.0300567 (PMC11185446; doi:10.1371/journal.pone.0300567)
Supplement: Attachment — Submitted filename: Response to Reviewers.pdf [file pone.0300567.s001.pdf]

October 4th, 2023

Editorial Department of PLOS ONE

Instituto Aragonés de Ciencias de la Salud

C/ de San Juan Bosco, 13, 50009 Zaragoza, Spain

Dear Dr. Andrea Tangherloni,

Thank you for your valuable feedback on our manuscript (PONE-D-23-13388) entitled "SinglePoint RNA, an user-friendly application implementing single cell RNA-seq analysis software" by Laura Puente and Luis del Peso.
Below, we provide a detailed response addressing the concerns raised by the referees, along with explanations of the modifications made to the manuscript text and figures.

On accordance with PLoS One format guidelines, we have made the following changes:
1. We have converted the figure file format to TIFF.
2. Author affiliation section has been revised.
3. The Data Availability statement has been expanded to specify in greater detail that the code and data used in this project is fully available through a GitHub repository at www.github.com/ScienceParkMadrid/SinglePointRNA

With regards to copyrighted images, we are submitting the signed permission form to display a screenshot of the software in Figure 1. Figure 5 has been changed and no longer displays an excerpt of the application's materials. Though I want to note that the image displayed in Figure 1 is not a reprint of another publication, but an edited screenshot taken in a personal computer after executing the code, which is distributed under a license that allows even patent and commercial use to third parties. Considering this, the wording added to the caption (*“Reprinted from [ref] under a CC BY license, with permission from [name of publisher], original copyright [original copyright year].”*) doesn't really match the context, so I'd like to ask if there was a better fitting formula to add to the figure's caption.

In response to the specific queries raised by Reviewer 1:

Major comments:

1 - We have replaced figure 5 for an overview of the app's structure.

2- We appreciate the referee's recognition of one of the key advantages of SinglePointRNA, which is its ability to simplify scRNAseq data analysis. We acknowledge the importance of providing practical examples to illustrate the convenience and utility of our method. To maintain the conciseness of the manuscript and to ensure that readers have easy access to these illustrative examples, we have chosen to include them in the User's Guide associated with the application.

The User's Guide serves as a comprehensive resource for users of SinglePointRNA, providing step-by-step instructions and examples for each analysis included in the package. By placing these examples in the guide rather than in the manuscript, we aim to offer users a more user-friendly and practical experience when working with the tool. This approach allows readers to access detailed guidance precisely when they need it, making it easier for them to apply SinglePointRNA effectively to their scRNAseq data analysis.

We believe that this strategy strikes the right balance between providing valuable examples and maintaining the manuscript's clarity and brevity. Users can conveniently access the User's Guide alongside the application to explore and understand the practical applications of SinglePointRNA.

3- We've added the list of analysis steps implemented in the Introduction (lines 25-28 of the manuscript)

4- SinglePointRNA is designed with a primary focus on enhancing user-friendliness and accessibility for inexperienced users, aiming to simplify the scRNAseq analysis process. It is important to clarify that the application does not aim to improve the speed or performance of the underlying tools it integrates.

We intentionally exclude certain options, such as parallelization, to prevent significant increases in memory requirements, which could pose challenges for users with limited computational resources.

In summary, SinglePointRNA prioritizes ease of use and accessibility for its target audience, and as such, its emphasis is on simplifying analysis steps rather than on optimizing performance speed.

Minor comments:
We have reviewed and revised the format and writing of the manuscript to improve clarity and readability.

Answer to queries from Reviewer 2

Comments on the manuscript

We greatly appreciate the referees' comments and their valuable insights into the comparison of SinglePointRNA, our Shiny-based R application, with existing tools such as ShinyCell, SCHNAPPs, and SEQUIN. We understand the importance of assessing SinglePointRNA's uniqueness and its contributions to the field of scRNAseq analysis.

ShinyCell (Bioinformatics, 37(19), 2021, 3374–3376) and similar tools, like scViewer (Cells. 2023 May 27;12(11):1489), primarily focus on visualizing processed single-cell data. They require preprocessed data as input and concentrate on visualization, making them complementary rather than direct alternatives to our app. SinglePointRNA, on the other hand, emphasizes comprehensive scRNAseq data processing, interactive visualization, and functional insights, thereby providing a broader utility.

Regarding SCHNAPPs (Journal of Immunological Methods 499 (2021) 113176), while it encompasses essential functions for complete scRNAseq data processing, it lacks certain features

that enhance biological interpretability. Specifically, SinglePointRNA introduces functional enrichment analysis of differentially expressed genes and automatic cell type imputation, both aimed at enhancing the biological relevance of the results. The incorporation of two novel metrics, co-clustering conservation and clustering uncertainty score, in conjunction with interactive Sankey flowcharts represents a key advancement, helping users make informed decisions on clustering resolution, a critical parameter for robust clustering outcomes. We consider these unique characteristics as defining improvements that set SinglePointRNA apart from existing tools.

Moreover, we acknowledge the recent publication of SEQUIN (2023, Cell Reports Methods 3, 100420) around the time of our manuscript submission to PLoS ONE. SEQUIN as SinglePointRNA was designed for non-bioinformaticians for complete raw scRNAseq analysis. Both tools provide dedicated graphs for parameter selection, and functional enrichment analysis of differentially expressed genes. However, an important distinction lies in the deployment approach: SinglePointRNA operates primarily as a stand-alone tool, offering local installation and operation flexibility. In contrast, SEQUIN leans towards web-based functionality, which may impose limitations on the scale of analyses. Furthermore, SinglePoint's novel metrics extend beyond providing results for a range of clustering resolution values, offering deeper insights into clustering performance.

In conclusion, we believe that SinglePointRNA, through its comprehensive processing capabilities, unique metrics, and flexibility in deployment, represents a significant advancement over existing tools like SCHNAPPs and SEQUIN. Its focus on facilitating sound clustering decisions and enriched biological interpretations positions it as an invaluable asset for the scRNAseq analysis community.

To acknowledge the existence of other Shiny-based scRNAseq tools we have included the following sentence in the discussion of our manuscript.

"We acknowledge the existence of other Shiny-based scRNAseq tools such as SCHNAPPs and SEQUIN. SCHNAPPs (Journal of Immunological Methods 499, 2021) presents comprehensive processing and analysis capabilities for raw scRNAseq data. However, SinglePointRNA, extends its utility by incorporating functional enrichment analysis and automatic cell type imputation to enhance biological insights. Notably, SinglePointRNA introduces novel metrics, co-clustering conservation and clustering uncertainty score, aiding users in selecting a meaningful clustering resolution, a critical parameter to get meaningful results. Furthermore, SEQUIN (Cell Reports Methods 3, 2023), a web-based tool, surfaced around the time of our manuscript submission. While SEQUIN shares many features with SinglePointRNA, such as parameter guidance and functional enrichment analysis, SinglePointRNA is uniquely positioned as a stand-alone tool with flexibility in local deployment. We appreciate these tools' contributions, yet SinglePointRNA's distinctive attributes underscore its value as an insightful alternative for scRNAseq analysis."

With regards to differential expression testing, as indicated in the manuscript (page 6 lines 147-148) SinglePointRNA uses the default DE test implemented in Seurat, which is based on the Wilcoxon Rank Sum test. We made this choice to take advantage of the flexibility of the test, because the input for our app is not restricted to particular protocols. Several of the tests available on Seurat are restricted to certain types of scRNA-seq data, such as UMI-tagged datasets for the methods "negbinom" and "poisson", and the more known methods in bulk RNA-seq, like DESeq2, which novice users could be more familiar with, are not as robust with the sample sizes typically compared in single-cell analysis.

As for future work planned, this project was developed as part of a pre-doctoral project that has already finished, so there are no plans established to keep expanding it, as long as I am concerned. However, this software was developed initially for the Genomis Unit at the Fundación Parque Científico de Madrid, which might have an interest in furthering development of the software, and in addition to that, it was published under an open source license that allows any member of the community to distribute their own modifications of the app, which we welcome with open arms.

All figures are now over 2000px wide.

Comments on the app.

1. The pop-up notification for the loading tab has been added. Clearing the data can be done by refreshing the app or the browser window, but for convenience we've added reset buttons on the loading and dataset merging tabs.

2. Regarding the issue of '-Inf' in the QC section, while we were unable to reproduce it, we have implemented controls to address it if it occurs. Regarding the request to exclude genes or features, such as mitochondrial genes or genes not detected in a minimum number of cells, which is a common requirement in single-cell analysis, we have a solution. In the 'Filter cells' tab, you will find the second-to-last option, which is designed to regress the effect of a variable on the dataset. This feature includes the expression levels of mitochondrial, ribosomal, and NRY genes, as well as genes used to estimate cell cycle phases. Regarding the ability to return to the original dataset and reapply the filters, please note that this would nearly double the memory requirements of the app to store a copy of the dataset. To provide users with insights into the filtering process, we've implemented a 'Check Filters' button. This feature allows users to assess how many cells might not pass the filter criteria before executing the task. We hope these features enhance the usability and efficiency of SinglePointRNA for your single-cell analysis needs.

3. On the clustering section, there isn't any specific reason to exclude number of neighbors, but since it isn't a direct parameter of the clustering algorithm, like clustering resolution, and it doesn't affect other steps like the number of principal components used, we just did not focus on it.

4. The main point of of the visualization section is to show how much 2D plots can differ (so novice users don't take them as direct measures of similitude) and to choose a convenient one to use as a base to plot results onto. Otherwise, the X-Y variables in each plot don't have to be directly comparable, much less by visual inspection alone.

5. For differential expression, as stated previously, the tests used is the Wilcoxon Rank Sum Test implemented in Seurat. Adding a color bar in heatmaps might be useful in very small datasets, but the function from Seurat that we're implementing in this section clips high expression values and sample sizes are usually too large to differentiate between color shades.

As it happens with the heatmaps, large sample sizes make adding a point layer to violin plots an issue. In this case, the dots tend to obscure the value distribution almost completely unless the opacity and dot size are turn to values so low that the whole dot layer becomes useless.

6. On the Altered Pathways section, group names have been added to legends.

7. The introduction to classification on the documentation has been revised.

Final statement

We extend our sincere gratitude to the reviewers for their meticulous evaluation of our package and manuscript, and for their valuable suggestions, which have significantly enhanced both SinglePointRNA and the manuscript. We believe that the manuscript is now prepared for publication in PLOS ONE.

Yours sincerely,

Laura Puente Santamaría

Instituto Aragonés de Ciencias de la Salud

C/ de San Juan Bosco, 13, 50009 Zaragoza, Spain

E-mail: laura.puentes@estudiante.uam.es

lpuente.iacs@aragon.es
