# Supplementary material for: SinglePointRNA, an user-friendly application implementing single cell RNA-seq analysis software
Source: PLoS One. 2024 Jun 18;19(6):e0300567. doi: 10.1371/journal.pone.0300567 (PMC11185446; doi:10.1371/journal.pone.0300567)
Supplement: Attachment — Submitted filename: Response to Reviewers.pdf [file pone.0300567.s002.pdf]

October 4th, 2023
Editorial Department of PLOS ONE

Instituto Aragonés de Ciencias de la Salud
C/ de San Juan Bosco, 13, 50009 Zaragoza, Spain

Dear Dr. Andrea Tangherloni,

Thank you again for the feedback on our manuscript (PONE-D-23-13388) entitled "SinglePoint RNA, an user-friendly application implementing single cell RNA-seq analysis software" by Laura Puente and Luis del Peso. Below, we provide a detailed response addressing the comments by the referees, along with explanations of the modifications made to the manuscript text and figures.

In response to the specific queries raised by Reviewer 1:

We have noticed that the figures are shown in a much lower resolution in the manuscript PDF file. The full sized images should be accessible through the link at the top right corner of each page. Nevertheless, we have increased both the resolution and size of all the figures.

In response to the specific queries raised by Reviewer 2:

The comparison between SinglePointRNA and other scRNA-seq related software has been expanded into its own section:

"The growing prominence of single-cell RNA sequencing (scRNAseq) has spurred the development of various tools dedicated to simplifying data visualization and analysis. Notable among these are ShinyCell, scViewer, and SCHNAPPs. Here, we discuss the distinctive features of SinglePointRNA in comparison to these tools, emphasizing its contributions to the state of the art in scRNAseq analysis.

ShinyCell, along with counterparts like scViewer, primarily focuses on sharing and visualizing preprocessed single-cell data. These tools serve as valuable complements to SinglePointRNA, as they demand preprocessed data as input and emphasize data visualization. In contrast, SinglePointRNA offers a more comprehensive approach, placing a strong emphasis on scRNAseq data processing, interactive visualization, and functional insights. This broader utility positions SinglePointRNA as a versatile tool for researchers seeking a comprehensive solution.

While SCHNAPPs encompasses essential functions for complete scRNAseq data processing, it lacks certain features that enhance biological interpretability. In comparison, SinglePointRNA introduces functionalities such as functional enrichment analysis of differentially expressed genes and automatic cell type imputation. In addition the incorporation of two novel metrics, co-clustering conservation and clustering uncertainty score, along with interactive Sankey flowcharts, represents a significant advancement. These features aid users in making informed decisions on clustering resolution, a crucial parameter for robust clustering outcomes. These unique characteristics set SinglePointRNA apart, adding depth and biological relevance to scRNAseq analysis.

During the manuscript submission, an additional tool named SEQUIN, designed for non-bioinformaticians conducting complete raw scRNAseq analysis and sharing similarities with SinglePointRNA, was introduced. Both tools facilitate parameter selection and functional enrichment analysis of differentially expressed genes through dedicated graphs. However, a crucial distinction arises in their deployment approaches. SinglePointRNA operates primarily as a stand-alone tool, allowing for local installation and operational flexibility. In contrast, SEQUIN leans towards web-based functionality, potentially imposing limitations on the scale of analyses. Additionally, SinglePointRNA's novel metrics offer deeper insights into clustering performance, providing researchers with a comprehensive toolkit for robust analysis.

Thus, SinglePointRNA stands out as a significant advancement in scRNAseq analysis, showcasing comprehensive processing capabilities, unique metrics, and flexible deployment options. Notably, its emphasis on empowering researchers with informed clustering decisions and enriched biological interpretations positions SinglePointRNA as an invaluable asset for the scRNAseq analysis community. The incorporation of novel metrics and a user-centric approach reflects its commitment to providing a robust toolkit for researchers seeking deeper insights into their single-cell RNA sequencing data."

Final statement

We want again to express our appreciation to the reviewers for their time and dedication in assessing our work. Your input has undoubtedly contributed to the overall enhancement of our manuscript, and we are grateful for your expertise and commitment to the peer review process.

Yours sincerely,

Laura Puente Santamaría

Instituto Aragonés de Ciencias de la Salud

C/ de San Juan Bosco, 13, 50009 Zaragoza, Spain

E-mail: laura.puentes@estudiante.uam.es

lpuente.iacs@aragon.es
